# Elevated IGFIR expression regulating VEGF and VEGF-C predicts lymph node metastasis in human colorectal cancer

**DOI:** 10.1186/1471-2407-10-184

**Published:** 2010-05-07

**Authors:** Chunhui Zhang, Li Hao, Liang Wang, Yichuan Xiao, Hailiang Ge, Zhenya Zhu, Yunbao Luo, Yi Zhang, Yanyun Zhang

**Affiliations:** 1 Shanghai Institute of Immunology, Institutes of Medical Sciences, Shanghai Jiao Tong University School of Medicine (SJTUSM) and Key Laboratory of Stem Cell Biology, Institute of Health Sciences, Shanghai Institutes for Biological Sciences, Chinese Academy of Sciences & SJTUSM, Shanghai, China; 2 Department of Surgery, Pudong New Area People's Hospital, Shanghai, China; 3 Clinical Laboratory, The First Affiliated Hospital of Anhui Medical University, Hefei, China; 4 Department of Surgery, First Affiliated Hospital of Soochow University, Suzhou, China; 5 Department of Internal Medicine, University of Michigan, Ann Arbor, USA

## Abstract

**Background:**

Insulin-like growth factor-I receptor (IGFIR) has been shown to regulate the tumor development. The objective of the current study is to determine the association of IGFIR with lymph node metastasis and to explore the related mechanism in human colorectal cancer in clinic.

**Methods:**

In a random series of 98 colorectal cancer patients, the expressions of IGFIR, vascular endothelial growth factor (VEGF) and VEGF-C were investigated by immunohistochemistry, and the association of these expressions with lymph node metastasis was statistically analyzed. The expressions of VEGF and VEGF-C in colorectal cancer cells stimulated with IGF-I were also examined by real-time quantitative reverse transcription-polymerase chain reaction and enzyme-linked immunosorbent assay.

**Results:**

Higher rates of IGFIR (46%), VEGF (53%), and VEGF-C (46%) expression were found in colorectal cancer tissues than in normal and colorectal adenoma tissues. These expressions were significantly associated with clinicopathologic factors and lymph node status. We also found the concomitant high expressions of IGFIR/VEGF (*P *< 0.001) and IGFIR/VEGF-C (*P *= 0.001) had a stronger correlation with lymph node metastasis than did each alone or both low expressions. In addition, IGF-I could effectively induce the VEGF and VEGF-C mRNA expression and protein secretion in colorectal cancer cells expressing IGFIR molecules. Moreover, Patients who had strong staining for IGFIR, VEGF and VEGF-C showed significantly less favorable survival rates compared with patients who had low staining for these molecules (*P *< 0.001). The survival rates of patients who were both high expression of IGFIR/VEGF and IGFIR/VEGF-C also were significantly lower compared with patients who were negative or one of high expression of these molecules (*P *< 0.001).

**Conclusions:**

Together the findings indicated for the first time that simultaneous examination of the expressions of IGFIR, VEGF and VEGF-C will benefit the diagnosis of lymph node metastasis in order to assay the prognosis and determine the treatment strategy in patients with colorectal cancer undergoing surgery.

## Background

Colorectal cancer is a major global health problem and the fourth most common cause of cancer death worldwide [1]. Distal metastasis that results from lymph node metastasis is one of the main causes of colorectal cancer death and is an unsolved difficult issue in clinical treatment. Accumulating evidence indicates that a variety of tumor systems including colorectal cancers express high levels of insulin-like growth factor-I receptor (IGFIR) [2,3], which initiates intracellular signaling cascades that enhance cell cycle progression and inhibit apoptosis [4], finally led to prosperity of cancer cells and increased tumor invasion [5,6]. Moreover, small-molecule inhibitors and antisense oligonucleotides to IGFIR could efficiently suppress the growth and proliferation of human cancer cells *in vitro *[7,8] and decrease lymph node metastasis in a mouse tumor model [9]. However, how IGFIR regulates tumor growth and lymph node metastasis in human colorectal carcinomas in clinic is still not well understood.

Angiogenesis has been known to play an important role in the development of tumor growth and lymph node metastasis. Vascular endothelial growth factor (VEGF) family is the most widely investigated and most specific regulator of angiogenesis, which consist of six members including VEGF-A, -B, -C, -D, -E and placenta growth factor. They potently increase vascular permeability and promote the formation of new blood vessels in tumors and thus are regarded as the main growth stimulatory factors in the tumor-related angiogenesis [10]. The prognostic value of high expression of VEGF (or VEGF-A) for lymph node metastasis has been demonstrated in various types of human cancer [10-12]. Most recent studies demonstrated that stimulation of IGFIR in colorectal cancer cells induced the expression of VEGF, which can further promote the progression of cancer by regulating the development of new blood vessels [13,14]. In comparison, blocking the IGFIR led to significant down-regulation of VEGF and inhibition of tumor growth and lymph node metastasis [7-9]. These observations suggest that IGFIR can promote the tumor growth and lymph node metastasis through the induction of VEGF.

The regional lymph nodes draining primary tumors are generally the first, and by far the most common, site of metastasis for some of the major human malignancies, and tumor cell dissemination to the regional lymph node was generally believed to be a passive process. Recent evidence suggests that tumor-derived VEGF-C and VEGF-D can stimulate *de novo *formation of intratumoral lymphatic capillaries (lymphangiogenesis), which raised the possibility that cells within primary tumors can contribute actively to lymphatic dissemination through the induction of lymphangiogenesis [15]. When overexpressed in breast cancer MCF-7 cells, VEGF-C promoted tumor lymphangiogenesis and tumor metastasis [16]. Notably, IGF-I has recently been demonstrated to be a positive regulator of VEGF-C expression through IGFIR signaling and implicated in the control of lymphatic metastasis [17].

Based on these observations, we wished to clarify the mechanism by which IGFIR regulate tumor growth and lymph node metastasis in human carcinomas. For this purpose, we investigate the expressions of IGFIR, VEGF and VEGF-C and the association of these expressions with lymph node metastasis in human colorectal cancer and relative cancer cell line. Our results show that the concomitant high expressions of IGFIR and VEGF or VEGF-C is positively correlated with lymph node metastasis in human colorectal cancer in a significant manner, which could provide valuable information in predicting and diagnosing lymph node metastasis.

## Methods

### Patients and specimens

Sections of paraffin-embedded tissue samples were provided by the Department of Pathology, Pudong New Area People's Hospital, Shanghai, China. They were obtained from patients with colorectal cancer and included 98 colorectal cancer tissue samples and 26 adjacent normal tissue samples. There were also 38 colorectal ademona tissues. Informed consent for sample collection and analysis of their tissue for research purposes in this study was obtained from the subjects. We retrospectively reviewed all patients who underwent curative-intent surgery and were randomly selected at the Department of Surgery from 2002 to 2005. Ethical approval was obtained to perform this research study from the Ethical Committee of Pudong New Area People's Hospital in Shanghai, China. Forty-eight of the 98 colorectal cancer patients had histologically confirmed lymph node metastasis. Tumors were staged according to the American Joint Committee on Cancer pathologic tumor-node-metastasis (TNM) classification. None of the patients had received pre-operative chemotherapy or radiotherapy therapy.

### Immunohistochemistry

Sections were subjected to routine deparaffinization and rehydration. Antigen retrieval was achieved by microwaving sections in 0.01 mol/L citrate buffer for 10 min and then cooling for 30 min. The endogenous peroxidase activity was inhibited by incubation with 3% hydrogen peroxide in methanol for 20 min and nonspecific binding was blocked by incubation with 5% bovine serum albumin in phosphate-buffered saline (PBS) at room temperature. After three PBS washes, the specimens were reacted overnight at 4°C with rabbit anti-human IGFIR polyclonal antibody (Abgent, San Diego, CA, USA), mouse anti-human VEGF monoclonal antibody (Lab Vision Corp., Fremont, CA, USA) or rabbit anti-human VEGF-C polyclonal antibody (Zymed Laboratories, Inc., South San Francisco, CA, USA). After incubation at 37°C with EnVision™ (DAKO, Hamburg, Germany) for 30 min, signal was developed with 3,3'-diaminobenzidine tetrahydrochloride in Tris-HCl buffer (pH 7.6) containing 0.3% hydrogen peroxide. The sections were then counterstained with hematoxylin and mounted. Negative controls were performed by replacing the specific primary antibody with normal mouse serum or PBS.

### Interpretation and evaluation of immunohistochemical results

Immunostaining was independently examined by two clinical pathologists who were unaware of the patient outcome. For each sample, five high-power fields (200×) were randomly selected. The percentage of positive tumor cells and staining intensity were assessed. The extent of the staining was categorized into five semiquantitative classes based on the percentages of positive tumor cells: 0 (≤ 5% positive cells), 1 (6%-25% positive cells), 2 (26%-50% positive cells), 3 (51%-75% positive cells) and 4 (>75% positive cells). The intensity of cytoplasmic and membrane staining was also determined semiquantitatively on a scale of 0-3 as follows: 0 (negative), 1 (weakly positive), 2 (moderately positive) and 3 (strongly positive). A consensus score was assigned for each section after discussion and careful review of all slides by the two pathologists.

Multiplication of the intensity and the percentage scores gave rise to the final staining score: 0 (negative), + (1-4), ++ (5-8), and +++ (9-12). For statistical analysis, tumors that scored 0 or +, which showed weak or moderate/strong immunoreactivity, were lumped into a low expression group and were compared to tumors with scores of ++ or +++ as the high expression group.

### Induction of VEGF and VEGF-C expression by IGF-I

COLO 205 cells, a colorectal cancer cells line, were plated in 96-well plates and cultured overnight at 37°C in Dulbecco's modified Eagle's medium (1 × 10^6^/ml) supplemented with 10% fetal bovine serum, penicillin (100 U/ml) and streptomycin (100 mg/ml). The medium was aspirated, and fresh, serum-free medium containing various concentrations of IGF-I (0, 20, 50, 100, 250 ng/ml) was then added into each well. After another 48-h culture at 37°C, the cells and supernatants were collected. Total RNA was extracted from the cells by Trizol reagents, and VEGF and VEGF-C mRNA levels were analyzed by real-time quantitative reverse transcription-polymerase chain reaction (qRT-PCR). VEGF and VEGF-C secretion in the culture supernatant was analyzed by enzyme-linked immunosorbent assay (ELISA).

### Statistical analysis

Chi-squared test and two-sided Fisher's exact test were applied to determine the strength of association between the categorical variables. The correlations between expression of IGFIR, VEGF and VEGF-C and several clinicopathologic parameters were assessed with the χ^2 ^test or the Spearman rank test. Survival rates were calculated using the Kaplan-Meier method, and significant differences in survival were determined by log-rank test. All statistical analyses were performed using a statistical software package (SPSS, Inc., Version 10.0, Chicago, IL, USA). All tests were two-sided, and *P *values < 0.05 were considered statistical significance.

## Results

### IGFIR, VEGF and VEGF-C expression in different colorectal tissues

**Figure 1:**
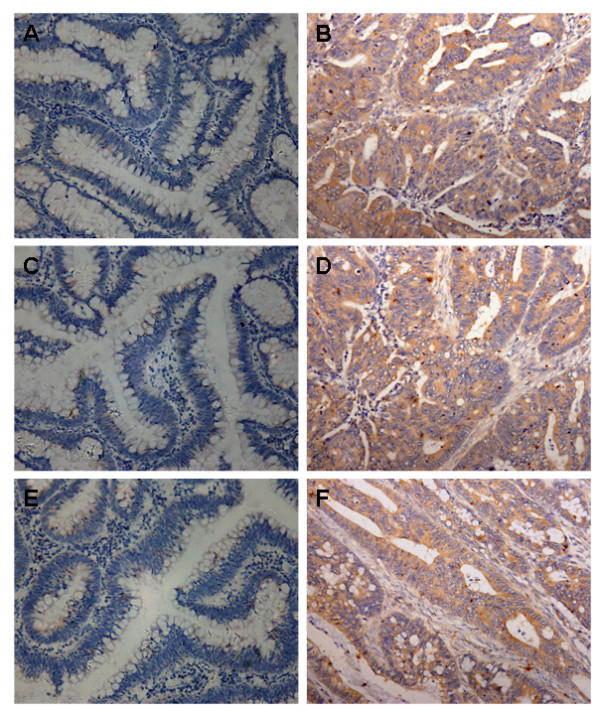
**Representative examples of immunohistochemical staining of IGFIR, VEGF, and VEGF-C in colorectal cancer**. Positive staining is shown in dark brown. Colorectal adenoma tissues were stained with IGFIR (A), VEGF (C), VEGF-C (E) as controls, and colorectal cancer tissues showed strong staining of IGFIR (B), VEGF (D), and VEGF-C (F). (magnification 200×)

IGFIR exhibited cytoplasmic and membrane staining. Two out of 26 normal tissues adjacent to the tumor cells showed weak IGFIR staining, and the rest were negative for IGFIR. Among the 38 colorectal adenoma tissues, 6 were negative for IGFIR, 3 exhibited strong positive staining and the rest had positive but weak IGFIR staining. The frequency of positive IGFIR staining in colorectal adenoma was higher than that in normal tissues. In striking contrast, all 98 colorectal cancer cases showed positive IGFIR staining with 45 cases (46%) being high expressors (Figure 1). As shown in Table 1, there are highly significant differences in terms of the number of cases with high IGFIR expression between the colorectal cancer and the normal group (*P *< 0.001) or between the cancer and the adenoma group (*P *< 0.001). VEGF and VEGF-C immunoreactivity, localized mainly to the cytoplasm, was weak in normal and adenoma samples, but their staining was much stronger in cancer tissues (53% for VEGF and 46% for VEGF-C; Figure 1).

**Table 1:** Expression of IGFIR in Different Colorectal Tissues.

	**Case No**.	IGFIR expression		*P *value
			
		Low or negative	High	
Colorectal tumor	98	53 (54%)	45 (46%)^a^	< 0.001 (a vs. b)
Colorectal adenoma	38	35 (92%)	3 (8%)^b^	0.265 (b vs. c)
Normal colorectal tissues	26	26 (100%)	0 (0%)^c^	< 0.001 (a vs. c)

### Association of IGFIR, VEGF and VEGF-C expression with clinicopathologic features

The clinical, epidemiologic, and histopathologic characteristics of patients with colorectal cancers, and the association of high expression of IGFIR, VEGF, and VEGF-C with clinicopathologic parameters were shown in Table 2. There was a statistically significant correlation between TNM stage and the level of IGFIR (*P *< 0.001), VEGF (*P *= 0.001) or VEGF-C (*P *= 0.006). The increased expressions were significantly associated with advanced histological grade (*P *< 0.001 for IGFIR, *P *= 0.007 for VEGF and *P *< 0.001 for VEGF-C). There were no statistically significant associations in the expression of these molecules with regard to patient age, gender, tumor position or the location of tumor infiltration.

**Table 2:** Correlation of IGFIR, VEGF and VEGF-C Expression with Clinicopathologic Features in Colorectal Tumor.

Clinicopathologic parameters	**Case No**.	IGFIR expression		*P *value	VEGF expression		*P *value	VEGF-C expression		*P *value
							
		Low	High		Low	High		Low	High	
Total cases	98	53 (54%)	45 (46%)		46 (47%)	52 (53%)		53 (54%)	45 (46%)	
Age										
≤50	25	14 (56%)	11 (44%)	0.824	11 (44)	14 (56%)	0.733	14 (56%)	11 (44%)	0.351
>50	73	39 (53%)	34 (47%)		35 (45%)	38 (55%)		33 (45%)	40 (55%)	
Gender										
Male	55	29 (53%)	26 (47%)	0.761	24 (44%)	31 (56%)	0.459	28 (51%)	27 (49%)	0.509
Female	43	24 (56%)	19 (44%)		22 (51%)	21 (49%)		19 (44%)	24 (56%)	
Tumor position										
Colon	44	20 (45%)	24 (55%)	0.122	19 (43%)	25 (57%)	0.501	23 (52%)	21 (48%)	0.440
Rectum	54	33 (61%)	21 (39%)		27 (50%)	27 (50%)		24 (44%)	30 (56%)	
Infiltration location										
Submucosa	30	19 (63%)	11 (37%)	0.109	17 (57%)	13 (43%)	0.180	15 (50%)	15 (50%)	0.052
Muscularis propria	6	1 (17%)	5 (83%)		1 (17%)	5 (83%)		0 (0%)	6 (100%)	
Outer layer	62	33 (53%)	29 (47%)		28 (45%)	34 (55%)		32 (52%)	30 (48%)	
TNM stage										
I	24	18 (75%)	6 (25%)	< 0.001	16 (67%)	8 (33%)	0.001	13 (54%)	11 (46%)	0.006
II	32	23 (72%)	9 (28%)		20 (63%)	12 (37%)		22 (69%)	10 (31%)	
III	36	10 (28%)	26 (72%)		8 (22%)	28 (78%)		10 (28%)	26 (72%)	
IV	6	2 (33%)	4 (67%)		2 (33%)	4 (67%)		2 (33%)	4 (67%)	
Histologic differentiation										
Poor	30	6 (20%)	24 (80%)	< 0.001	7 (23%)	23 (77%)	0.007	5 (17%)	25 (63%)	< 0.001
Moderate	44	30 (68%)	14 (32%)		24 (55%)	20 (45%)		24 (55%)	20 (45%)	
Good	24	17 (71%)	7 (29%)		15 (63%)	9 (37%)		18 (75%)	6 (25%)	
Vascular invasion										
Negative	49	34 (69%)	15 (31%)	0.002	34 (69%)	15 (31%)	< 0.001	32 (65%)	17 (35%)	0.001
Positive	49	19 (39%)	30 (61%)		12 (24%)	37 (76%)		15 (31%)	34 (69%)	
Lymph node metastasis										
Negative	50	36 (72%)	14 (28%)	< 0.001	30 (60%)	20 (40%)	0.008	32 (64%)	18 (36%)	0.001
Positive	48	17 (35%)	31 (65%)		16 (33%)	32 (67%)		15 (31%)	33 (69%)	

Abbreviations: IGFIR, insulin-like growth factor-I receptor; VEGF, vascular endothelial growth factor; TNM, tumor-node-metastasis.

### Association of high expression of IGFIR with lymph node metastasis

Vascular invasion directly influences the status of lymph node metastasis. Out of 49 cancer samples positive for vascular invasion, there were 30 cases (61%) showing high expression of IGFIR, whereas only 15 cases (31%) for 49 samples of negative vascular invasion (*P *= 0.002). There was also a significant correlation between IGFIR expression levels and lymph node metastasis. Thirty-one cases (65%) out of 48 samples positive for lymph node metastasis showed high expression of IGFIR in both colorectal cancer tissues and related metastatic lymph nodes, whereas only 14 cases (28%) for 50 samples of negative lymph node metastasis (*P *< 0.001; Table 2). These data suggested that IGFIR may play an important role in the promotion of lymph node metastasis in human colorectal cancer.

### Association of single or combined high expression of IGFIR and VEGF or VEGF-C with lymph node metastasis

We then analyzed the combined effects of IGFIR and VEGF or VEGF-C in human colorectal cancer. Statistic analysis showed a significant association of increased expression of IGFIR, VEGF, and VEGF-C with lymph node metastasis in patients suffering from colorectal cancer. The incidence of lymph node metastasis tended to be higher in cases with high rather than low expression of IGFIR, VEGF and VEGF-C, respectively (*P *< 0.01; Table 2). We further compared the correlation of colorectal cancer lymph node metastasis with combined high expression of both IGFIR and VEGF or of both IGFIR and VEGF-C. The incidence of lymph node metastasis was significantly higher in patients with tumors highly expressing both IGFIR and VEGF (75%) than those of patients with tumor highly expressing only one or none of the molecules (34%). Similarly, higher rate of lymph node metastasis was observed in patients with tumors highly expressing both IGFIR and VEGF-C (70%) than those of patients with tumors highly expressing only one or none of the molecules (36%). Notably, when compared with both low or one of high expression of these markers, the concomitant high expressions of IGFIR and VEGF or IGFIR and VEGF-C was statistically significantly associated with lymph node metastasis in patients with colorectal cancer (Table 3; *P *< 0.001 for IGFIR/VEGF and *P *= 0.001 for IGFIR/VEGF-C).

**Table 3:** Correlation of Combined High Expression of IGFIR and VEGF or VEGF-C with Lymph Node Metastasis.

	Lymph node metastasis		*P *value
		
	Negative	Positive	
**IGFIR/VEGF**			
Both low or one of high expression	41	21	< 0.001
Both high expression	9	27	
**IGFIR/VEGF-C**			
Both low or one of high expression	39	22	0.001
Both high expression	11	26	

### Interrelationship between IGFIR and VEGF or VEGF-C expression

We further examined whether the expression of IGFIR was significant correlated with the expression of VEGF or VEGF-C in human colorectal tumor. Among the 45 colorectal tumor samples with high expression of IGFIR, 36 cases showed high expression of VEGF and also 36 cases for VEGF-C. In addition, statistical analysis indicated that high expression of IGFIR was positively correlated with the high expression of VEGF (Table 4; r = 0.497, *P *< 0.001) and VEGF-C (Table 4; r = 0.516, *P *< 0.001).

**Table 4:** Correlation Analysis between High Expression of IGFIR and VEGF or VEGF-C in Colorectal Tumor.

IGFIR	VEGF		VEGF-C	
	
	Low	High	Low	High
Low	37	16	38	15
High	9	36	9	36
	r = 0.497		r = 0.516	
	*P *< 0.001		*P *< 0.001	

Correlation coefficients (r) were calculated by Spearman rank correlation analysis.

### IGF-I induced VEGF and VEGF-C expression in colorectal cancer cells

**Figure 2:**
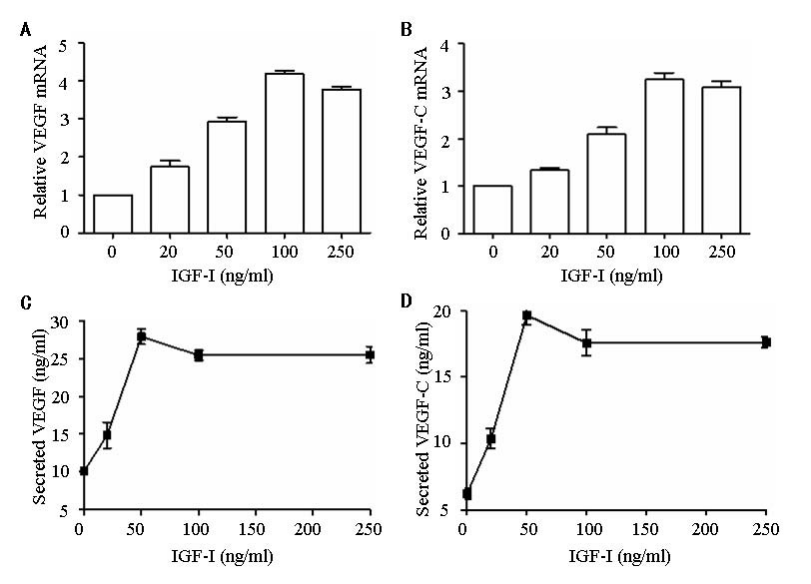
**Induction of VEGF and VEGF-C by IGF-I in colorectal cancer cell**. COLO 205 cells were incubated with various concentrations of IGF-I for 48 h at 37°C. The expression of VEGF (A) and VEGF-C (B) was analyzed by qRT-PCR in COLO 205 cells, and normalized to endogenously expressed β-actin in the same sample. VEGF (C) and VEGF-C (D) protein secretion in the cell culture supernatant was quantified using ELISA. Results are expressed as the mean ± SD of triplicate cultures and are represented of 3 independent experiments.

To investigated the possible the relationship of IGFIR expression and VEGF or VEGF-C in human colorectal carcinoma, a colorectal cancer cell line COLO 205, which expressed IGFIR molecules, were used and stimulated with various dose of IGF-I. Expression of VEGF and VEGF-C mRNA was then determined by real-time qRT-PCR, and secreted VEGF and VEGF-C were analyzed by ELISA. As shown in Figure 2, there was a dose dependent increase of VEGF and VEGF-C mRNA and protein upon the stimulation of IGF-I, suggesting that the expression of VEGF and VEGF-C could be induced in human colorectal cancer through IGFIR signal.

### Prognostic impact of IGFIR, VEGF and VEGF-C expression

**Figure 3:**
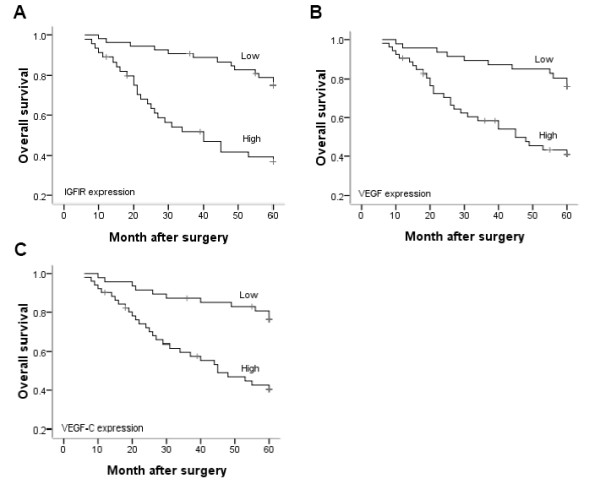
**Kaplan-Meier analysis of overall survival for IGFIR, VEGF and VEGF-C expression**. Survival curves for patients with colorectal cancer were stratified by the expression of IGFIR (A), VEGF (B) and VEGF-C using the Kaplan-Meier method. Patients who had strong staining for IGFIR, VEGF and VEGF-C showed significantly less favorable survival rates compared with patients who had low staining for these molecules (*P *< 0.001).

**Figure 4:**
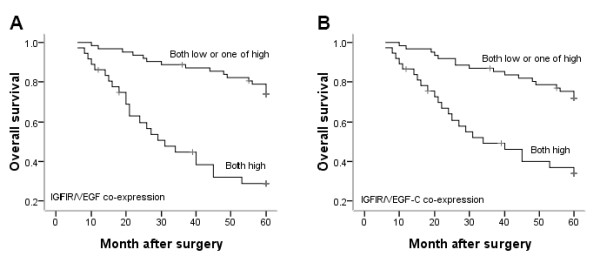
**Kaplan-Meier analysis of overall survival for IGFIR/VEGF and IGFIR/VEGF-C co-expression**. Survival rates were evaluated according to the combination of IGFIR/VEGF and IGFIR/VEGF-C co-expression. The group of patients with both high expression of IGFIR/VEGF and IGFIR/VEGF-C exhibited the poorer prognosis (*P *< 0.001).

The prognostic impacts of IGFIR, VEGF and VEGF-C expression were analyzed by using Kaplan-Meier survival curves. Patients who had strong IGFIR, VEGF and VEGF-C staining showed a significantly lower survival rate compared with patients who had low staining (Figure 3, *P *< 0.001). Survival rates also were evaluated according to combinations of IGFIR/VEGF and IGFIR/VEGF-C co-expression. Patients who were both high expression of IGFIR/VEGF and IGFIR/VEGF-C had unfavorable prognoses, and patients who were negative or one of high expression of these molecules had more favorable prognoses (Figure 4, *P *< 0.001).

## Discussion

In this study, we found that simultaneously high expression of IGFIR and VEGF or VEGF-C were valuable predictive biomarker of lymph node metastasis in human colorectal cancer. Furthermore, statistical analysis and *in vitro *results indicated that elevated IGFIR expression was responsible for the upregulation of VEGF and VEGF-C, which then promote the lymph node metastasis in human colorectal cancer.

Malignant tumor metastasis is the result of a series of complex processes that depend on multiple and interactive factors. IGFIR signaling has recently been inferred to play a role in tumor growth and the invasive and metastatic ability in various carcinomas *in vitro *and in animal models [18]. VEGF, which stimulates the migration and proliferation of endothelial cells, has been well recognized as a crucial regulatory factor in tumor angiogenesis. A number of studies have demonstrated that VEGF can induce tumor growth and promote lymphatic metastasis [19,20]. Lymphangiogenic factors, such as VEGF-C and VEGF-D, have been show to stimulate tumor lymphangiogenesis and metastasis to regional lymph nodes by interacting with their receptor VEGF receptor 3 (VEGFR-3). High levels of VEGF-C and VEGF-D can promote tumor growth and lymph node metastasis in human colorectal cancer. Moreover, strong expression of VEGF-C and VEGF-D was significantly linked to a trend for decreased survival in colorectal cancer patients [21-25]. Our results in pathology indicated that high expression of each of these three markers was much more frequent in human colorectal cancer tissues than in normal tissues or non-tumor lesions. While the expression of IGFIR, VEGF and VEGF-C correlated with tumor TNM stage, the increased expression of IGFIR, VEGF, and VEGF-C also correlated with histological grade. Our observations suggest that these molecules play important roles in mediating the tumor progression of malignancy. Furthermore, we found that the expression of IGFIR, VEGF, and VEGF-C significantly correlated with lymph node status. Thus, investigating the expression and relationship of these markers may elucidate the mechanism by which regulates lymph node metastasis in human colorectal cancer.

It was implied that IGFIR play a role in tumor growth and lymph node metastasis in various cancers cells [18]. We found that the cases of high expression of IGFIR were more in the patients with positive lymph node metastasis than that with negative lymph node metastasis. Moreover, there is a statistically significant correlation of high expression of IGFIR in colorectal cancer tissue with lymph nodes metastasis, suggesting that patients suffering from colorectal cancer with high expression of IGFIR have stronger lymph node metastatic possibility.

Recently, some of *in vitro *experiments indicated that IGFIR can increase VEGF expression and stimulate tumor angiogenesis in pancreatic carcinoma cells [26,27]. Moreover, inhibiting IGFIR signaling can not only down-regulate the expression of VEGF, but also decrease the number of tumor-related blood vessels, increase cancer cell apoptosis and lose lymph node metastatic ability to distal organs [28]. It is likely that tumor progression and metastasis could be promoted by IGFIR via the induction of angiogenesis. However, although the evidences for IGFIR-VEGF interaction have been put forward in the results *in vitro*, there is still lack of evidence currently available on the biological relationship between these two molecules in human colorectal cancer in clinic. In our study, we showed a significant correlation between the high expressions of IGFIR and VEGF in human colorectal cancer samples. In addition, the concomitant high expressions of both molecules were more predictive of lymph node involvement as compared with that of a single marker high expression or both low expressions. Furthermore, IGF-I could effectively stimulate VEGF mRNA expression and protein secretion in human colorectal cancer cells expressing IGFIR molecules, which implied VEGF receptor VEGFR could also be up-regulated to promote the induction of angiogenesis, Therefore, VEGF and its receptor VEGFR may be a downstream molecule that is regulated by IGFIR signaling, and they may have synergistic effects in colorectal cancer growth and lymph node metastasis.

Metastatic tumor spread through the blood or lymphatic vessels occurs in most forms of human cancer, with regional lymph node metastasis often being the most important prognostic factor for carcinoma patients [29]. It was reported that IGF-I can act as a direct lymphangiogenic factor through the activation of intracellular signal components, such as Akt, Src, and extracellular signal-regulated kinase in tumor lymphatic endothelial cells [30]. Furthermore, M-27 cells, a Lewis lung carcinoma subline, transfected with human IGFIR cDNA expressed high levels of VEGF-C mRNA and protein in response to IGF-I [17]. These results implied that IGFIR may stimulate the expression of VEGF-C, and indirectly modulate the potential of tumor lymph node metastasis. By immunohistochemical staining, we found that in human colorectal cancer samples that expressed high levels of both IGFIR and VEGF-C, 70% were positive for lymph node metastasis, and the co-expression of IGFIR and VEGF-C in the tumor correlated with an increased incidence of lymph node metastasis. In addition, our data showed that IGFIR high expression was positively correlated with VEGF-C high expression, and the expression of VEGF-C in mRNA and protein level also could be effectively induced by IGF-I in human colorectal cancer cells expressing IGFIR molecules, implying VEGF-C receptor VEGFR-3 could also be stimulated to promote lymphangiogenesis. Thus, IGFIR and VEGF-C may also play a synergistic role in human colorectal cancer metastasis, and this process may partly contribute to the upregulation of VEGF-C by IGFIR.

PI3K has been identified as a major transducer of the IGFIR signal in various cellular systems. Among others, its activity was shown to be critical for cell survival, a function mediated through Akt and Bax activation, and it was implicated in mitogenesis, protein synthesis, and differentiation. The degree to which different cells use common pathways to convey the IGFIR signals may be cell context dependent [17]. Thus, IGFIR signaling elevated VEGF and VEGF-C expression could be mediated by PI3K pathway, which may be possible mechanisms underlying induction of VEGF and VEGF-C by IGF-I in human colorectal cancer cell lines or tumor samples.

Predictive prognostic markers in colorectal cancer were studied, and a few statistically significant associations between the studied markers and longterm prognosis were found. In the current study, we found a clear and significant correlation between high IGFIR, VEGF and VEGF-C expression and lymph node metastasis in human colorectal cancer. In addition, high expression of these molecules in colorectal cancer patients showed significantly less favorable survival rates. The combination analysis of IGFIR/VEGF and IGFIR/VEGF-C co-expression demonstrated a negative impact on prognosis. Therefore, these findings may enable more an accurate assessment of the predictive prognosis of patients with colorectal cancer.

## Conclusion

The present study demonstrates that IGFIR, VEGF, and VEGF-C are highly expressed in human colorectal cancer and that their expression is interrelated. In addition, the concomitant high expressions of IGFIR/VEGF or IGFIR/VEGF-C were significantly associated with lymph node metastasis and negatively impacted on survival rate in human colorectal cancer. Moreover, VEGF and VEGF-C could be effectively induced by IGF-I in human colorectal cancer cells expressing IGFIR molecules. These observations suggest that lymph node metastasis in human colorectal cancer could be promoted by elevated IGFIR expression via upregulation of VEGF and VEGF-C. Overall, combined analysis of IGFIR and VEGF or VEGF-C will prove to be invaluable for predicting lymph node metastasis in human colorectal cancer. Inhibition of IGFIR/IGF-I based on this mechanism may develop an effective treatment for human colorectal cancer. This study would provide a novel insight into diagnosis of lymph node metastasis and therapeutic strategy of tumor in human colorectal cancer.

## Abbreviations

IGFIR: insulin-like growth factor-I receptor; VEGF: vascular endothelial growth factor; TNM: tumor-node-metastasis; PBS: phosphate-buffered saline; qRT-PCR: quantitative reverse transcription-polymerase chain reaction; ELISA: enzyme-linked immunosorbent assay.

## Competing interests

The authors declare that they have no competing interests.

## Authors' contributions

CZ: designed the study, carried out the experiments and statistical data analysis; LH and LW: carried out the experiments and interpreted the data; YX: carried out the experiments, interpreted the data and wrote the manuscript; HG: supported with expertise in molecular biology techniques and in data interpretation; ZZ and YL: analysed the immunohistochemical data and critically revised the manuscript; YiZ: data analysis and critically revised the manuscript; YaZ: conceived the study, participated in study design and coordination, molecular and data analysis, data interpretation and drafting of the manuscript. All authors read and approved the final manuscript.

## Pre-publication history

The pre-publication history for this paper can be accessed here:

http://www.biomedcentral.com/1471-2407/10/184/prepub
